# Eukaryotic translation initiation factor 3 subunit B could serve as a potential prognostic predictor for breast cancer

**DOI:** 10.1080/21655979.2021.2017567

**Published:** 2022-01-18

**Authors:** Shaoran Song, Jie Liu, Miao Zhang, Xiaoqian Gao, Wei Sun, Peijun Liu, Yaochun Wang, Juan Li

**Affiliations:** a Center for Translational Medicine, The First Affiliated Hospital of Xi’an Jiaotong University, Xi’an, China; b The Key Laboratory for Tumor Precision Medicine of Shaanxi Province, The First Affiliated Hospital, Xi’an Jiaotong University, Xi’an, Shaanxi China

**Keywords:** Breast cancer, translation initiation factor eif3b, prognosis, proliferation

## Abstract

The EIF3 gene family is essential in controlling translation initiation during the cell cycle. The significance of the EIF3 subunits as prognostic markers and therapeutic targets in breast cancer is not yet clear. We analyzed the expression of EIF3 subunits in breast cancer on the GEPIA and Oncomine databases and compared their expression in breast cancer and normal tissues using BRCA data downloaded from TCGA. Then we performed clinical survival analysis on the Kaplan–Meier Plotter database and clinicopathologic analysis on the bc-genexMiner v4.1 database. And EIF3B was chosen for mutation analysis via the Cancer SEA online tool. Meanwhile, we performed the immunohistochemical assay, real-time RT-PCR, and Western blotting to analyze EIF3B expression levels in breast cancer. An EIF3B knockdown and a negative control cell line were conducted for MTT assay and cell cycle analysis to assess cell growth. Specifically, the results of TCGA and online databases demonstrated that upregulated EIF3B was associated with poorer overall and advanced tumor progression. We also confirmed that EIF3B was more highly expressed in breast cancer cells and tissues than normal and correlated with a worse outcome. And knockdown of EIF3B expression inhibited the cell cycle and proliferation. Furthermore, EIF3B was highly mutated in breast cancer. Collectively, our results suggested EIF3B as a potential prognostic marker and therapeutic target for breast cancer.

## Introduction

Breast cancer is a malignancy worldwide and has the highest prevalence among women [1]. As a heterogenic disease, breast cancer is highly associated with the abnormal expression of cellular molecules. Although the concepts in diagnosis and treatment have been taking account of the heterogeneity of breast cancer over the past 10–15 years, there are still insufficient ways to improve breast cancer prognosis of OS (overall survival) and RFS (relapse-free survival) [2]. Therefore, it is crucial to find out the potential biomarkers associated with the occurrence and prognosis of breast cancer and investigate the underlying molecular mechanisms.

*EIF3* (Eukaryotic Translation Initiation Factor 3) is the largest translation initiation complex. It is comprised of 13 subunits in mammals (a, b, c, d, e, f, g, h, i, k, l, m), corresponding to five core subunits in the budding yeast Saccharomyces cerevisiae (*a/Tif32, b/Prt1, c/Nip1, i/Tif34, g/Tif35*) [3,4]. Increasing evidence indicates that *EIF3* plays a unique role in the regulation of translation initiation process [5], re-initiation on downstream cistrons [6–9], translation termination [10], recycling ribosomal [11,12], and the readthrough of the programmed stop codon [13,14]. Owing to the essential function of *EIF3* in various physiological processes, it has been demonstrated its dysregulation is associated with various pathological conditions, especially in the incidence, development, and prognosis of different human cancers. Recent studies have indicated that the upregulation of *EIF3A*/ *B/ C/ H*/ *I/ M*, or downregulation of *EIF3E* and *EIF3F* were related to metastasis in several cancers [15–18]. However, the treatment and prognostic role of individual *EIF3* subunits in breast cancer has not been elucidated clearly.

Therefore, this study aimed to evaluate the biological functions and prognostic roles of EIF3 subunits in breast cancer. We examined the transcriptional expression levels, clinical prognostic significance, and survival value of individual *EIF3* subunits in breast cancer by performing comprehensive bioinformatics analysis on several large online databases. And *EIF3B* was selected for investigating the role in the diagnosis of breast cancer. Combined with experimental verification, we also confirmed the crucial function of *EIF3B* in breast cancer. We provided a potential molecular mechanism regulating tumor progression specific biomarker for breast cancer, which was improved than previous studies [19,20].

## Materials and Methods

### Data acquisition and Bioinformatics Analysis

The RNA-sequencing data and clinical information of patients with BRCA (Breast invasive carcinoma) were downloaded in HTSeq-FPKM format from TCGA (The Cancer Genome Atlas) (https://portal.gdc.cancer.gov/) and GTEx (Genotype-Tissue Expression) (https://commonfund.nih.gov/GTEx/) databases (n = 1222). Then, the HTSeq-FPKM format data was converted to TPM (transcripts per million reads) format data and these values were scaled using the equation: log_2_ (TPM+1) [21]. Moreover, the RNA-sequencing data in TPM format were downloaded from TCGA and GTEx for differential expression analysis of GTEx.

### Cell culture and transfection

MDA-MB-231 (Shanghai Institute of Biochemistry and Cell Biology) and MCF7 cells (gift from Dr Jianmin Zhang) were cultured in a DMEM medium with 10% FBS. BT549 and T47D cells were cultured in 1640 medium with 10% FBS. All cells were cultured in incubators with 5% CO_2_ and 37°C.

Two EIF3B siRNA (small interfering RNA) constructs were used to knockdown EIF3B expression in cells in vitro. These EIF3B siRNA constructs and a negative control siRNA were obtained from GenePharma Company as follow: EIF3B-1, 5′- GGAAGCAGAUGGAAUCGAUTT −3′ and 5′- AUCGAUUCCAUCUGCUUCCTT −3′; EIF3B-2, 5′- CCCUGGAUACGCUUAGCAUTT −3′ and 5′- AUGCUAAGCGUAUCCAGGGTT −3′. Used to transiently transfect into MDA-MB-231 cells in 3.5-cm plates for 48–72 h [the transfected with 100 pmol siRNA and 4 μl Lipo8000™ Transfection Reagent (#C0533, Beyotime, Nanjing, China) in 125 μl of Opti-MEM medium (Invitrogen)].

### Real-time RT-PCR and Western blotting

The real-time RT-PCR and Western blotting was performed as previously described [22]. The antibody of EIF3B was purchased from Santa Cruz Biotechnology. Primers of EIF3B were obtained from Takara as follows: Former: 5′- AGGTACCTGTGGATGTGGTCGAG-3′; Later: 5′- CCGTGCAGCACAGCAAACTTA −3′.

### Immunohistochemistry staining

The immunohistochemistry staining was performed as the previous described [19]. Tissues were fixed in 10% neutralized formaldehyde for 24 h and embedded in paraffin. The Primary antibodies mentioned above were applied overnight at 4°C. A biotinylated secondary antibody (ZSGB-Bio, Beijing, China) was used to detect the primary antibody, followed by incubation with diaminobenzidine before counterstaining with hematoxylin. Finally, the sections were dehydrated in graded ethanol and transparentized in xylene. Images were taken by a Leica SCN400 slide scanner (Leica).

### MTT assay and cell cycle analysis

The MTT assay was performed as previously described [22]. Cells transfected with *EIF3B* siRNA constructs and a negative control siRNA for up to 48 h were seeded into 48-well plates at a density of 5 × 10^4^ cells/well and transiently transfected with. At different periods, 50 μl MTT solution (5 mg/mL) was added to each well and cells were cultured for another 4 h at 37°C. After adding 375 μl Formazan solution to each well, the optical density (OD) at 490 nm was measured using a microplate reader (PerkinElmer, Germany). Each experiment was in triplicate and repeated at least three times.

Seventy-two hours after transfection, cells were harvested and subjected to flow cytometry. The samples were incubated overnight in 70% ethanol at 4°C and then incubated with 50 μg/mL propidium iodide (PI; Sigma, st. Louis, MO) and 10 μg/mL ribonuclease a (Sigma, st. Louis, MO, MO) in the dark for 30 min. The samples were then analyzed by flow cytometry (BD Biosciences, San Jose. CA, USA) to quantify the DNA content and the results were analyzed by FlowJo 10.

### Gene expression profiling interactive analysis (GEPIA) database

GEPIA (http://gepia.cancer-pku.cn/) is a publicly accessible online database that provides data from the cancer genome atlas (TCGA; https://tcga- data.nci.nih.gov/tcga/) and the genotype-tissue expression project (GTEx; https://www.gtexportal.org/home/index.html) [23]. In the current study, we used GEPIA to compare the differential expression of EIF3 graphically between breast cancer specimens and normal tissues.

### Oncomine and Breast cancer gene-expression Miner (bc-genexMiner v4.1)

The Oncomine database (http://www.oncomine.org), as a bioinformatics tool, is widely used for cancer transcriptome data collection, standardization, analysis and delivery [24]. Here, the transcriptional level of the *EIF3* complex was analyzed in breast cancer.

Bc-GenExMiner v4.1(http://bcgenex.centregauducheau.fr) contains 36 renowned genome datasets, which support its three basic bioinformatic functions: expression, prognosis and correlation [25]. The published annotated genomic data was last updated in December 2017, which can be used to evaluate the predictive significance of target genes in breast cancer and provides valuable prognostic biomarkers. Using Welch’s test, we investigated the correlation between the RNA levels of *EIF3* complex and different clinicopathological parameters of breast cancer.

### Kaplan–Meier Plotter database

The Kaplan-Meier Plotter tool (www.kmplot.com) is based on meta-analysis to detect the survival of different cancers [26]. To analyze the OS (overall survival), PFS (progression-free survival) and PPS (post-progression survival) of patients with different kinds of breast cancer, all the samples were divided into two groups by median transcriptional expression of *EIF3* subunits and assessed by a Kaplan-Meier survival plot. We also analyze the prognostic significance of *EIF3* subunits in different Lymph node statuses and different types of clinicopathologic classifications in breast cancer. The results of the Kaplan-Meier survival plot were shown with 95% CI (confidence intervals), HR (hazard ratio), and log-rank P-value. The various sample sizes for each survival analysis are due to the uncertain availability of all patient gene expression levels. A log P-value <0.01 was considered statistically significant.

### cBioPortal and Human Protein Atlas

As a comprehensive online database, the cBioPortal (http://www.cbioportal.org/) for Cancer Genomics provides comprehensive analysis based on multidimensional cancer genomic data [27]. Using the online instructions of cBioPortal, we calculated the CNV (copy number variation), mutations, and the summary of *EIF3B* in breast cancer.

The Human Protein Atlas (http://www.proteinatlas.org/) provides over 10 million IHC images and 82,000 high-resolution IF (immunofluorescence) images of tissue microarrays, which contains sections from 46 normal human tissues and more than 20 human cancers labeled with antibodies against more than 11,000 human proteins [28,29]. The staining intensity is classified as negative, weak, moderate or intense, based on the laser power and detector gain parameters used for image capture and in combination with the image’s visual appearance. Protein expression score determination was described as previous [30].

## Results

The *EIF3* gene family, as a crucial complex in affecting the occurrence and progression of cancers, may function in breast cancer treatment and prognosis in the future. Here, we aimed to evaluate the biological functions and prognostic roles of *EIF3* subunits in breast cancer. Based on the online datasets, we performed a comprehensive bioinformatics analysis based on the online datasets to assess the transcriptional expression levels, clinical prognostic significance, and survival value of individual *EIF3* subunits in breast cancer. Furthermore, *EIF3B* was selected for mutation analysis and experimental verification also suggested the important role of *EIF3B* in affecting breast cancer progression through cell cycle regulation.

### The transcriptional levels of EIF3 complex in breast cancer and para-carcinoma tissues

**Figure 1.**
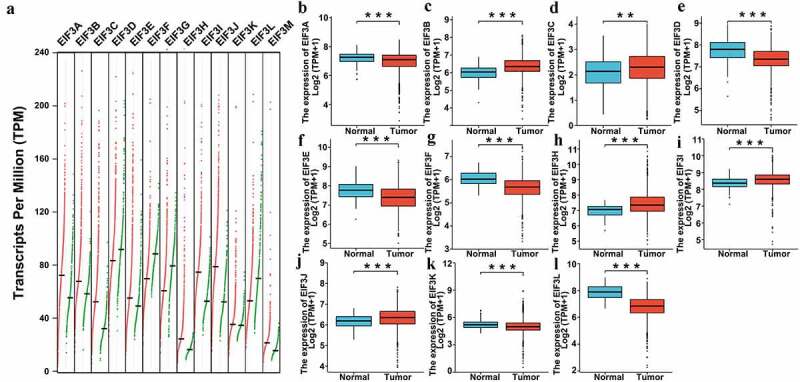
**The transcriptional levels of EIF3 in breast cancer and para-carcinoma tissues (GEPIA and TCGA)**. (a) Gene Expression Profile (dot plots) of the transcription levels of EIF3 family members in breast cancer and para-carcinoma tissues analyzed using GEPIA. Right, breast cancer tissues; left, para-carcinoma tissues. (b-l) Boxplot results of the expression levels of EIF3 family members in breast cancer analyzed using data from TCGA. Left box, normal samples; right box, tumor samples. *** means P < 0.001.

To determine the diagnostic role of the EIF3 complex in breast cancer, we used the GEPIA online database and BRCA (Breast Invasive Carcinoma) data downloaded from TCGA (The Cancer Genome Atlas) to compare the transcriptional levels of EIF3 complex expression in different cancer types. As shown in Figure 1, significantly higher transcriptional levels of *EIF3A/ B/ C/ E/ H/ I/ J/ M* and lower transcriptional levels of *EIF3D/ F/ G/ L* in breast cancer compared to para-carcinoma tissues (Figure 1(a), *p < 0.001*) and TCGA both indicated significantly higher expression of *EIF3B/ C/ H/ I/ J* and lower *EIF3D/F /L* in breast cancer tissues (Figure 1(b-l), *P < 0.001*).

Next, we investigated the transcriptional level of individual *EIF3* subunits in different breast cancer datasets (Table 1). In Finak’s dataset, *EIF3B* was overexpressed in invasive breast carcinoma versus normal tissue with a fold change of 3.280; as for ductal breast carcinoma, *EIF3B* only increased with a fold of 1.33, which suggested a higher expression of *EIF3B* was accompanied by tumor progression [31]. In addition, Finak et al. also showed that *EIF3C* (*P < 0.001*, fold change = −9.750), *EIF3E* (*P < 0.001*, fold change = −42.681), *EIF3F* (*P < 0.001*, fold change = −47.085), and *EIF3I* (*P < 0.001*, fold change = −6.013) were decreased in several kinds of breast cancer compared to standard samples [31]. Additionally, Ramaswamy et al. [32] reported that the low mRNA level of *EIF3F* was found in different kinds of breast cancer (*P < 0.001*, fold change = −3.372). A low expression level of *EIF3G* was found in ductal breast carcinoma in the Richardson dataset [33]. Furthermore, Ma et al. [34] demonstrated that the mRNA expression of *EIF3D* (*P < 0.001*, fold change = −3.372), *EIF3G* (*P < 0.001*, fold change = −2.559), and *EIF3L* (*P < 0.001*, fold change = −2.213) were significantly decreased in ductal breast carcinoma in situ epithelial when compared to adjacent breast tissues. The same trend of *EIF3L* was observed in Mucinous Breast Carcinoma (*P < 0.001*, fold change = −2.208), Invasive Ductal Breast Carcinoma (*P < 0.001*, fold change = −2.112) according to the data from TCGA, as well as in ductal breast carcinoma (*P < 0.001*, fold change = −2.071) based on the data from the study of Richardson et al [33]. Consistent with the results of GEPIA and TCGA, it indicated significantly higher *EIF3B* expression and lower *EIF3D/ F/ L* in breast cancer tissues.

**Table 1.** The transcription level of EIF3 Expression change in between different types of breast cancer and normal breast tissues (oncomine database)

Gene	Types of cancer vs normal	Fold change		t-test	P-value	Dataset
*EIF3B*	Invasive Breast Carcinoma Stroma	3.280		15.936	1.19E-20	Finak et al [31]
	Invasive Breast Carcinoma	1.249		7.612	2.93E-10	Gluck et al [62]
	Ductal Breast Carcinoma	1.418		1.906	0.053	Hedenfalk et al [63]
	Ductal Breast Carcinoma	1.330		2.830	0.009	Sorlie et al [64]
*EIF3C*	Ductal Breast Carcinoma in Situ Epithelia	−9.750		−16.155	1.79E-18	Finak et al [31]
	Ductal Breast Carcinoma	−1.627		−1.198	0.138	Turashvili [65]
*EIF3D*	Ductal Breast Carcinoma in Situ Epithelia	−2.323		−5.285	2.04E-5	Ma et al [34]
	Invasive Ductal Breast Carcinoma Epithelia	−2.040		−4.189	2.82E-4	
	Ductal Breast Carcinoma	−1.797		−6.650	2.84E-8	Richardson et al [33]
	Invasive Breast Carcinoma Stroma	−1.478		−6.251	6.05E-6	Finak et al [31]
*EIF3E*	Lobular Breast Carcinoma	−42.681		−22.757	1.63E-28	Finak et al [31]
	Invasive Lobular Breast Carcinoma	−1.524		−6.999	4.92E-10	TCGA
	Invasive Ductal and Lobular Carcinoma	−1.325		−7.489	2.60E-9	
	Lobular Breast Carcinoma	−1.776		−5.373	2.13E-4	
*EIF3F*	Invasive Breast Carcinoma Stroma	−47.085		−23.776	1.42E-10	Finak et al [31]
	Breast Cancer	−3.372		−2.916	0.007	Ramaswamy [66]
	Male Breast Carcinoma	−2.180		−5.456	2.08E-4	TCGA
*EIF3G*	Ductal Breast Carcinoma in Situ Epithelia	−2.559		−6.053	4.20E-6	Ma et al [34]
	Ductal Breast Carcinoma	−2.047		−6.083	2.56E-7	Richardson et al [33]
*EIF3I*	Invasive Breast Carcinoma Stroma	−6.013		−16.311	9.48E-18	Finak et al [31]
	Invasive Ductal Breast Carcinoma Stroma		−1.816	−3.219	0.003	
*EIF3L*	Ductal Breast Carcinoma in Situ Epithelia	−2.213		−6.525	4.88E-5	Ma et al [34]
	Mucinous Breast Carcinoma	−2.208		−5.640	1.43E-4	TCGA
	Invasive Ductal Breast Carcinoma	−2.112		−15.335	3.63E-27	
	Ductal Breast Carcinoma	−2.071		−9.018	7.42E-12	Richardson et al [33]

### Relationship between the transcriptional level of EIF3 and the clinicopathological grade of breast cancer

**Figure 2.**
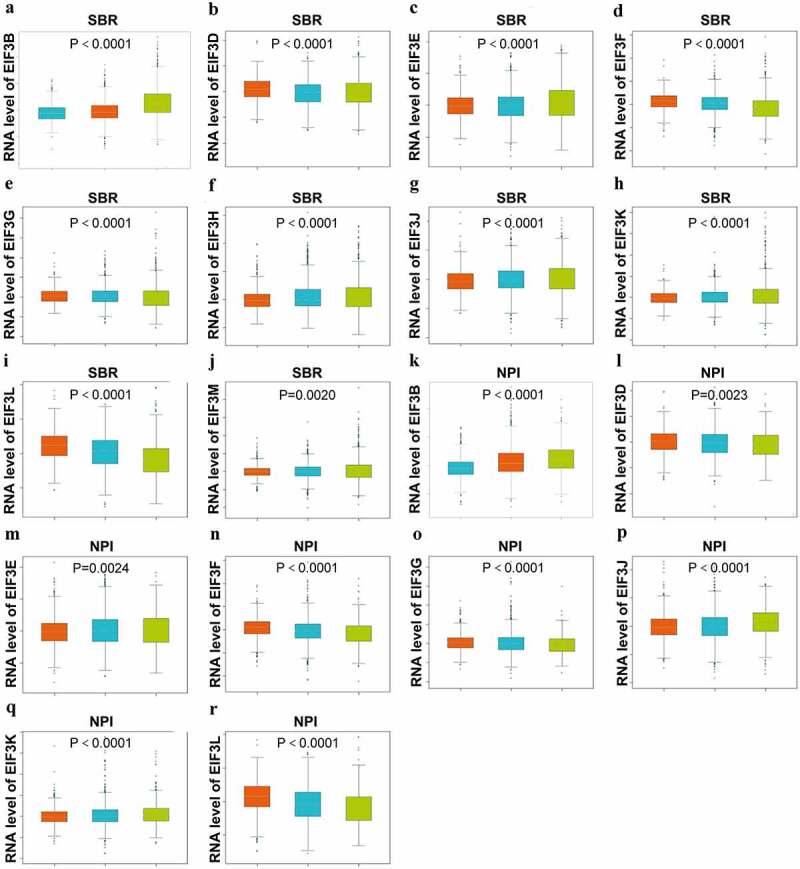
**Relationship between the transcriptional level of EIF3 and the clinicopathological parameters of breast cancer (bc-GenExMiner v4.1)** Boxplot results of the expression levels of EIF3 family members in different grades of SBR and NPI of breast cancer. Left, grade I; middle, grade II;right, grade III.

Through the bc-GenExMiner v4.1, we found that the transcriptional level of the *EIF3* complex was highly associated with breast cancer according to the SBR (Scarff–Bloom–Richardson) grade criterion and the NPI (Nottingham Prognostic Index) grade criterion. As the grades of SBR and NPI were higher, the mRNA expression level of *EIF3D/ F/ G/ L* were downregulated (Figure 2(b), *p < 0.0001*; Figure 2(d), *p < 0.0001*; Figure 2(e), p < *0.0001*; Figure 2(i), *p < 0.001*; Figure 2(l), *p < 0.05*; Figure 2(n), *p < 0.001*; Figure 2(o), *p < 0.001*; Figure 2(r), p < *0.001*); while *EIF3B*/ *E*/ *J*/ *K* represented the opposite trends (Figure 2(a), *p < 0.0001*; Figure 2(c), *p < 0.0001*; Figure 2(g), p < *0.001*; Figure 2(h), p < *0.0001*; Figure 2(k), *p < 0.0001*; Figure 2(m), *p < 0.01*; Figure 2(p), p < *0.0001*; Figure 2(q), p < *0.0001*). Also, *EIF3H* and *EIF3M* were highly expressed in higher SBR grades (Figure 2(f), *p < 0.0001*; Figure 2(j) *p < 0.01*). The comparison between the transcriptional expression of other individual *EIF3* subunits and the SBR and NPI grade showed statistical significance (*P < 0.01*), as shown in Table S1. These results showed that upregulated *EIF3B/ E/ J/ K* and downregulated *EIFD/ F/ L* was significantly associated with advanced clinicopathological grades.

### Correlation between the transcriptional level of EIF3 and survival

**Figure 3.**
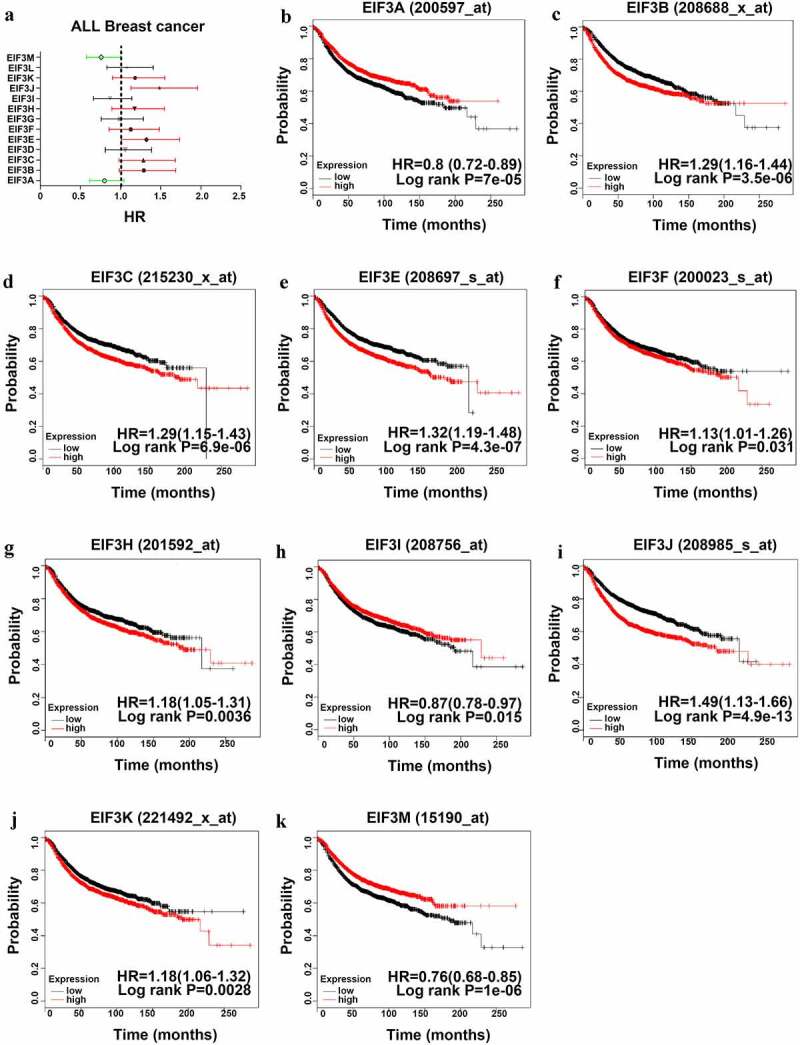
**The Prognostic Value of EIF3 in Breast cancer (RFS in Kaplan–Meier Plotter)** (a) Prognostic HRs of individual EIF3 members in all breast cancer. (b-k) Survival curves of EIF3A (Affymetrix ID: 200597_at), EIF3B (Affymetrix ID: 208688_x_at), EIF3C (Affymetrix ID: 215230_x_at), EIF3E (Affymetrix ID: 208697_s_at), EIF3F (Affymetrix ID: 200023_s_at), EIF3H (Affymetrix ID: 201592_at), EIF3I (Affymetrix ID: 208756_at), EIF3J (Affymetrix ID: 208985_s_at), EIF3K (Affymetrix ID: 221494_x_at), EIF3M (Affymetrix ID: 215190_at). p < 0.05.

The Kaplan-Meier Plotter was performed to investigate the association between the transcriptional levels of *EIF3* subunits and the prognosis of patients with breast cancer. A higher mRNA expression of *EIF3A/ I/ M* showed better RFS in patients with breast cancer (Figure 3(b), HR = 0.8, 95% CI: 0.72–0.89, *P < 0.001*; Figure 3(h), HR = 0.87, 95% CI: 0.78–0.97, *P < 0.05*; Figure 3(k), HR = 0.76, 95% CI: 0.68–0.85, *P < 0.001*). In contrast, low *EIF3B /C /E/ F/ H/ J/ K* expression were related to poorer prognosis in breast cancer (Figure 3(c), HR = 1.29, 95% CI: 1.16–1.44, *P < 0.001*; Figure 3(d), HR = 1.29, 95% CI: 1.15–1.43, *P < 0.001*; Figure 3(e), HR = 1.32, 95% CI: 1.19–1.48, *P < 0.001*; Figure 3(f), HR = 1.13, 95% CI: 1.01–1.26, *P < 0.001*; Figure 3(g), HR = 1.18, 95% CI: 1.05–1.31, *P < 0.001*; Figure 3(i), HR = 1.49, 95% CI: 1.33–1.66, *P < 0.001*; Figure 3(j), HR = 1.18, 95% CI: 1.06–1.32, *P < 0.01*). As for other *EIF3* subunits, there was no statistical difference between their transcriptional expression levels and the prognosis of breast cancer patients. The OS, DMFS, and PPS for each *EIF3* subunit in each dataset were also shown in Table S2. Furthermore, we investigated whether the mRNA expression levels of individual *EIF3* subunits had a close relationship with their clinical prognosis for patients with different clinicopathologic classifications, including nodal status and histologic grade, as shown in Table S3 and S4. Here, the results suggested that only *EIF3B* as a prognostic biomarker was significantly related to poor survival of breast cancer.

### The Mutation Analysis of EIF3B in Breast Cancer

**Figure 4.**
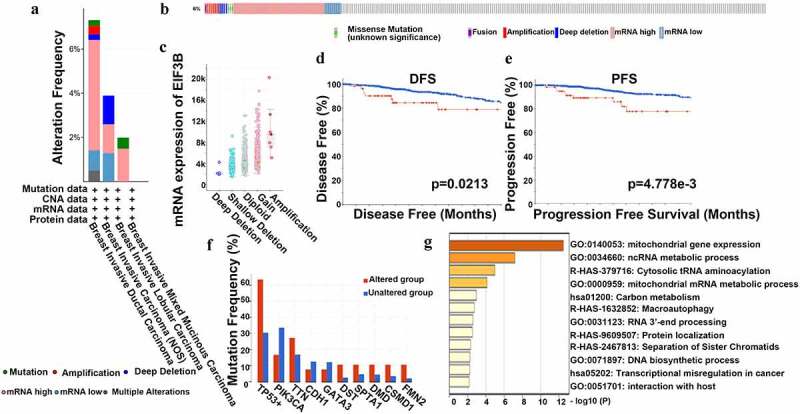
**The Mutation Analysis of EIF3B in Breast Cancer (cBioPortal)** (a-c) Genetic alteration of EIF3B in breast cancer. (d) Kaplan-Meier plots comparing OS in cases with/without EIF3B alterations. (e) The 50 most frequently altered neighbor genes with/without EIF3B alterations.

Based on the analysis results above, we discovered that the transcriptional level of *EIF3B* in breast cancer tissues was higher than that of para-carcinoma tissues, and over-expressed *EIF3B* indicated poor survival showed a consistent trend among the above databases and suggested it as an oncogene. Thus, we selected the *EIF3B* subunit to identify the frequency of genetic alterations in *EFI3B* among breast cancer patients using the cBioPortal database. 66 (6%) samples of 1084 patients with breast cancer showed significant alterations in *EIF3B*, including fusion, amplification, diploid, deep deletion, gain, missense mutation, and shallow deletion (Figure 4(a-b)). More than half of the alterations were mRNA high expression and five percent of alterations were gene amplification (Figure 4(c)). The association between the alteration of *EIF3B* and breast cancer prognosis was also examined. A higher mutation of *EIF3B* was accompanied by worse DFS (*P < 0.05*) and RFS (*P < 0.01*) in breast cancer (Figures 4(d) and 4(e)), while the OS showed no significance. Furthermore, the 50 most frequently altered neighbor genes were identified, including *TP53, PIK3CA, TTN, CDH1, GATA3, DST, SPTA1, DMD, CSMD1*, and *FMN2* (Figure 4(f)). Therefore, the results confirmed that the higher *EFI3B* mutation leads to a poor prognosis of breast cancer, and the mutation-related genes were associated with important physiological processes, which may explain the reason why the increased *EIF3B* leads to tumorigenesis.

### The Expression pattern of EIF3B in Breast Cancer

**Figure 5.**
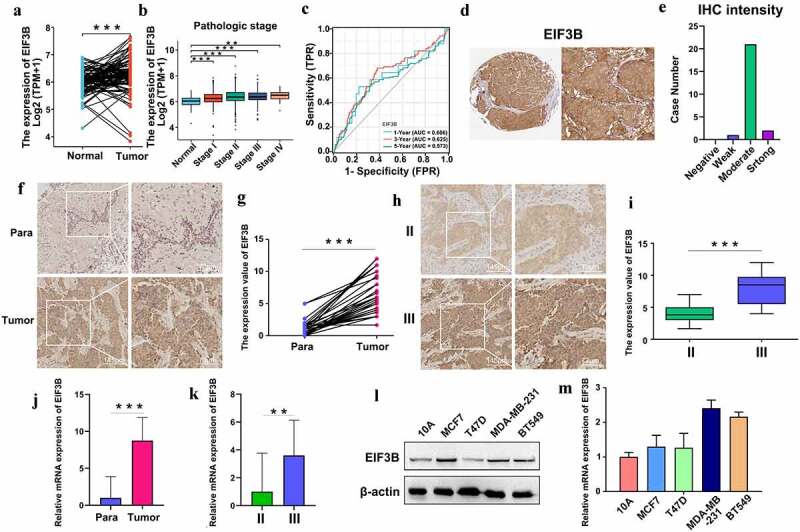
**The Expression pattern of EIF3B in Breast Cancer (TCGA, HPA, IHC, RT-PCR and WB)** (a) EIF3B mRNA expression in 112 pairs of breast cancer tissues and matched non-cancer tissues from TCGA data. Left, breast cancer tissues; right, non-cancer tissues. (b) EIF3B mRNA expression with pathologic grade of breast cancer. (c) ROC curve of EIF3B mRNA expression in breast cancer for 1-, 3-, and 5-year survival. (d) Protein expression levels of EIF3B across clinical specimens of breast cancer. (e) Bar charts for IHC staining intensities of EIF3B (23 patients). (f) The EIF3B protein expression pattern in breast cancer tissues and adjacent normal breast tissues (IHC). (g) Paired samples comparison chart for the expression levels of EIF3B in 30 pairs of breast cancer tissues and matched normal tissues. Left box, normal samples; right box, tumor samples. (h) The EIF3B protein expression pattern in different breast cancer pathologic stages (IHC). (i) Boxplot results of the expression levels of EIF3B in different pathologic stages of breast cancer tissues. Left box, Stage II; right box, Stage III. (j) The relative mRNA expression of EIF3B in breast cancer tissues and adjacent normal breast tissues (RT-PCR). Left, normal samples; right, tumor samples. (k) The relative mRNA expression of EIF3B in different pathologic stages of breast cancer tissues (RT-PCR). Left, Stage II; right, Stage III. (l-m) The protein and mRNA expression levels of EIF3B in different breast cancer cell lines (WB and RT-PCR). *** means P < 0.001; ** means P < 0.01.

Based on the BRCA data from the TCGA database, we confirmed the higher expression of *EIF3B* compared in 112 pairs of BRCA tissues and matched non-cancer tissues (Figure 5(a)), and the *EIF3B* expression level was significantly related to the pathologic stage (Figure 5(b)). Moreover, the ROC (receiver operating characteristic) curves associated with the AUC (area under the curve) values for 1-, 3-, and 5-year survival were 0.606, 0.625, and 0.573, respectively (Figure 5(c)). The protein expression of *EIF3B* was also analyzed in clinical specimens from the Human Protein Atlas, which demonstrated that *EIF3B* presented moderate protein expressions (Figure 5(d-e)). To verify the expression pattern of *EIF3B*, we performed IHC (immunohistochemistry), Western blotting, and real-time RT-PCR. The *EIF3B* protein expression pattern in breast cancer tissues and adjacent normal breast tissues was tested by IHC (Figures 5(f) and 5(h)). It showed that *EIF3B* was more highly expressed in breast cancer tissues than matched paraneoplastic tissues and more highly expressed in tissues of high pathological grade than those of low pathological grade (Figures 5(g) and 5(i)), which was consistent with the trend of mRNA expression in tissues from breast cancer patients (Figure 5(j-k)). Moreover, the protein and mRNA expression of *EIF3B* were examined in different types of breast cancer cell lines, including human breast epithelial cell lines (MCF10A), luminal breast cancer cells (MCF7), and T47D), and triple-negative breast cancer cells (MDA-MB-231 and BT549) (Figure 5(l-m)). It showed that expression of *EIF3B* was higher in MDA-MB-231 and BT549 than in MCF10A, MCF7, and T47D. The results confirmed that the upregulated EIF3B in breast cancer cells and tissues was highly correlated with advanced pathologic stages and poor survival.

### The Correlation and enrichment analysis of EIF3B in Breast Cancer

**Figure 6.**
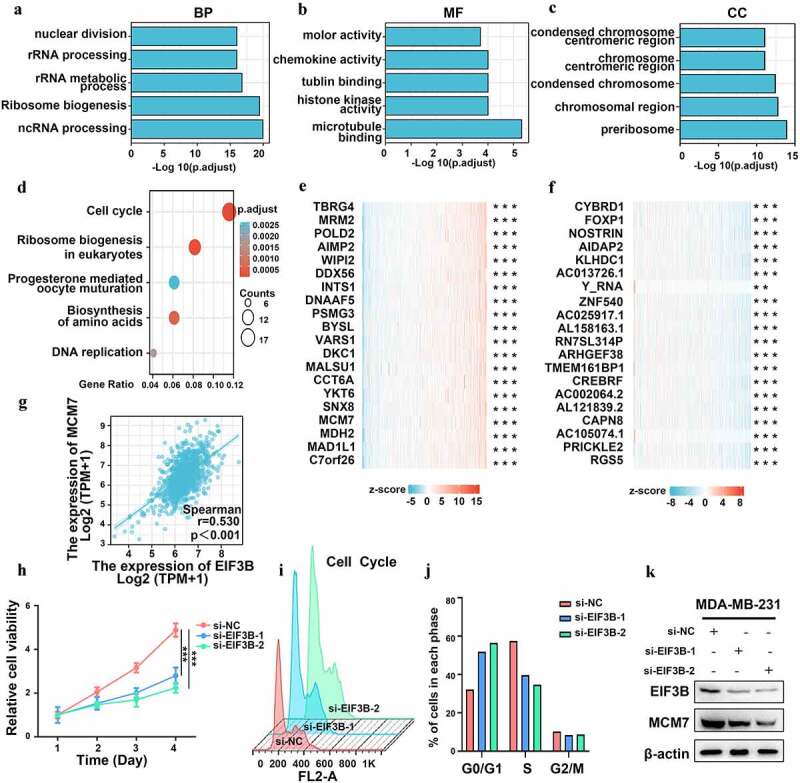
**The Correlation and enrichment analysis of EIF3B in Breast Cancer (GO, KEGG, MTT assay, and WB)** (a–c) Significant Gene Ontology terms of the top 200 genes most positively associated with EIF3B, including biological processes (BP), molecular function (MF), and cell component (CC). (d) Significant KEGG pathways of the top 200 genes most positively associated with EIF3B. (e-f) Top 20 genes most positively and negatively associated with FCGBP are shown in a heatmap. (g) Correlation between EIF3B mRNA expression and MCM7 mRNA expression using data from TCGA. (h) Cell viability using the MTT assay. The percentage of cell viability was calculated from the OD values of the test groups normalized to the control group. (i-j) Representative percentage of cells in the G0/G1, G2/M and S phases was detected by flow cytometric analysis. (k) Correlation between EIF3B protein expression and MCM7 protein expression using Western blot.

To explore the function and related pathways of *EIF3B*, we conducted a correlation analysis of *EIF3B* and other genes in breast cancer using TCGA data (Figure 6). As shown in Figure 6(a-c), we selected the top 200 genes for Functional enrichment and GO (Gene Ontology) analysis, including BP (biological processes), MF (molecular function), and CC (cell component). In addition, KEGG (Kyoto Encyclopedia of Genes and Genomes) pathway analysis indicated an enrichment and crosstalk of the top 200 genes in the cell cycle, ribosome biogenesis in eukaryotes, progesterone-mediated oocyte maturation, biosynthesis of amino acids, as well as DNA replication (Figure 6(d)). The top 20 genes most positively and negatively associated with *EIF3B* are shown in a heatmap, respectively (Figure 6(e-f)). Based on the results above, we noticed that *EIF3B* was significantly associated with MCM7, the DNA replication licensing factor, which regulates the cell cycle and cell proliferation [35,36]. Then, the correlation between *EIF3B* and *MCM7* was evaluated (Figure 6(g), r = 0.530, P < 0.001). Moreover, after the knockdown of *EIF3B* expression, we found cell viability and the G1/S transition were inhibited in the EIF3B-downregulated group compared with that of the control group (Figure 6(h-j)). Interestingly, the expression of MCM7 decreased after the knockdown of *EIF3B* expression (Figure 6(k)), indicating that *EIF3B* might inhibit breast cancer cell growth by inhibiting the G1/S transition of the cell cycle. Together, our results implied that *EIF3B* could affect tumor progression by regulating the cell cycle and proliferation.

## Discussion

*EIF3* complex, as a crucial complex in the process of translation initiation, its abnormal expression is closely related to various pathological processes [18,37–40]. Recent studies have shown the irregulated *EIF3* expression in various kinds of developmental diseases and human cancers, such as HNSCC (head and neck squamous cell carcinoma) [38], GBC (gallbladder cancer) [37], OV (ovarian cancer) [39], BRCA [18], HCC (hepatocellular carcinoma) [40] and so on. However, there have not been enough studies to elucidate the diverse roles of individual *EIF3* subunits in breast carcinoma. In the current research, the expressions and mutations of individual *EIF3* subunits in patients with breast cancer were comprehensively analyzed based on gene transcriptional expression or variation copy number online. It is hoped that our findings will facilitate future in-depth research and provide novel diagnostic and prognostic markers and therapeutic targets for breast cancer.

Based on our results, EIF3B was chosen as a more meaningful molecular target for further research in breast cancer. *EIF3B*, also referred to as MIP Prt1 homolog, *EIF3*S9, EIF-3-Eta, HPrt1, P110, and P116, has been previously known as the master scaffolding subunit in translation initiation. Cell proliferation, migration, and invasion are inhibited when *EIF3B* is down-regulated in cells of OV, STAD (gastric cancer), NSLC (non-small cell lung cancer), ESCC (esophageal squamous cell carcinoma), and ccRCC (clear cell renal cell carcinoma) [41–45]. However, very little was found in the literature on the detailed function of *EIF3B* in breast cancer. The present study was designed to determine the prognostic role of *EIF3* subunits. Our bioinformatics research and experimental results both revealed that the expression of *EIF3B* was higher in breast cancer cells and tissues than in normal. Consistently, the mRNA expression of *EIF3B* was positively related to tumor stage, SBR, and NPI grade in breast cancer. Furthermore, high expression and mutation of *EIF3B* were positively associated with poor prognosis in breast cancers. Finally, GO analysis and KEGG pathway analysis suggested that *EIF3B* was correlated to cell cycle and proliferation, and this was confirmed by the results of the MTT assay and cell cycle analysis. In accordance with the present results, previous studies have demonstrated that *EIF3B* downregulation suppresses cell proliferation, migration and invasion, and induces cell apoptosis by blocking the β-catenin pathway in endometrial cancer [46] or the PI3K/AKT/mTOR pathway in gastric cancer [41]. Moreover, we noticed *EIF3B* exhibited a high positive correlation with *MCM7* among the top 20 most positive genes, and our experimental results also confirmed that knockdown of *EIF3B* led to inhibition of *MCM7*, which has not been described previously. As an important license factor in DNA replication initiation [47,48], plenty of studies have verified the crucial role of *MCM7* in tumor proliferation [35], cancer stemness [36,49], migration and invasion [50]. Therefore, a possible explanation for the role of *EIF3B* in regulating breast cancer might be that it affects the expression of *MCM7* to block the G1/S transition of the breast cancer cell cycle and cell proliferation. This finding, while preliminary, suggests that translational factors could directly regulate replicational factors in tumor progression. Thus, further research should be undertaken to investigate the exact molecular mechanism of *EIF3B* in regulating replication licensing factors expression.

As for other EIF3 subunits, several studies have investigated their roles in tumors, including bladder cancer and pancreatic cancer [51,52], OV [39], cervical cancer [53], LUAD (lung adenocarcinoma) [54], and HCC [55]; few studies mention their role in breast cancer. We revealed that higher mRNA expression of *EIF3A* was accompanied by better RFS in breast cancer, which is not consistent with its function as a proto-oncogene as reported [51,52]. Deregulated EIF3C and EIF3D suppressed proliferation and promoted apoptosis in breast cancer, indicating that EIF3C and EIF3D act as oncogenes in breast cancer [56], whereas our findings showed that EIF3C and EIF3D were downregulated in breast cancer and that high expression of EIF3C and EIF3D was negatively related to worse outcomes in various types of breast cancer. According to the database, we discovered a lower expression of *EIF3E* and *EIF3F* in breast cancer. However, their mRNA levels were negatively related to the RFS of patients with breast cancer, which is contrary to their tumor-suppressing effect, as Shi et al. reported [57]. Proto-oncogenic *EIF3H* and *EIF3I* likely regulate the protein level of downstream factors to facilitate the developmental process of cancer [58,59]. Here, we determined that there was not a significant difference in the comparison between the expression of EIF3H and NPI grades, and that up-regulated *EIF3I* was correlated with a better outcome for patients with breast cancer, which made their function remain controversial. Inconsistent with its cancer-promoting role [60,61], K-M Plotter demonstrated that higher transcriptional levels of *EIF3M* led to better RFS (particularly those classified as grade III). Moreover, there is currently no in-depth research about the effects of *EIF3G*/ *J/ K/ L* on breast cancer. In our report, *EIF3G/ K/ L* was down-regulated in breast cancer tissues compared with normal tissues, while *EIF3J* presented the opposite trend. And a higher transcriptional level of *EIF3J* and *EIF3K* was associated with advanced pathologic grades and poor outcomes for breast cancer patients. Thus, a more in-depth study is needed to clarify their role in breast tumorigenesis.

## Conclusion

In summary, this study comprehensively evaluated the transcriptional level and prognostic significance of *EIF3* subunits in breast cancer. We also identified the crucial role of *EIF3B* in breast cancer progression by regulating the cell cycle and proliferation. Therefore, *EIF3B* could be a prognostic biomarker and might be a potential therapeutic target in BRCA.

## Supplementary Material

Supplemental Material

## Data Availability

All data generated or analysed during this study are included in this published article [and its supplementary information files].

## Highlight

1. Upregulated EIF3B was associated with poorer overall and tumor progression.

2. EIF3B was associated with cell cycle and proliferation.

3. EIF3B maybe a novel biomarker for breast cancer diagnosis and prognosis.

## Abbreviations

EIF3: translation initiation factor 3; BRCA: Breast invasive carcinoma; TCGA: The Cancer Genome Atlas; GTEx: Genotype-Tissue Expression; bc-genexMiner v4.1: Breast cancer gene-expression Miner; RT-PCR: Real-time PCR; small interfering RNA: siRNA; CI: confidence intervals; HR: hazard ratio; SBR: Scarff–Bloom–Richardson; NPI: Nottingham Prognostic Index; GEPIA: Gene Expression Profiling Interactive Analysis Platform; cBio Cancer Genomics Portal: cBioPortal; GO: Gene Ontology; BP: biological processes; MF: molecular function; CC: cell component; KEGG: Kyoto Encyclopedia of Genes and Genomes; HNSCC: neck squamous cell carcinoma; GBC: gallbladder cancer; OV: ovarian cancer; HCC: hepatocellular carcinoma; STAD: gastric cancer; NSLC: non-small cell lung cancer; ESCC: esophageal squamous cell carcinoma; ccRCC: clear cell renal cell carcinoma; LUAD: lung adenocarcinoma.
