# Supplementary material for: Eukaryotic translation initiation factor 3 subunit B could serve as a potential prognostic predictor for breast cancer
Source: Bioengineered. 2022 Jan 18;13(2):2762–76. doi: 10.1080/21655979.2021.2017567 (PMC8974155; doi:10.1080/21655979.2021.2017567)
Supplement: Supplemental Material [file KBIE_A_2017567_SM8035.zip › supplementary/ST1.pdf]

Supplementary Table 1. The results of Dunnett-Tukey-Kramer's test for pairwise comparison in SBR criterion and NPI criterion.

Gene	Pairwise comparison of SBR	P-value	Pairwise comparison of NPI	P-value
EIF3A	SBR 3 < SBR 1	<0.10	NPI 3 < NPI 1	<0.05
	SBR 3 < SBR 2	<0.10	NPI 2 = NPI 1	>0.10
	SBR 2 = SBR 1	>0.10	NPI 3 = NPI 2	>0.10
EIF3B	SBR 2 > SBR 1	<0.0001	NPI 2 > NPI 1	<0.0001
	SBR 3 > SBR 1	<0.0001	NPI 3 > NPI 1	<0.0001
	SBR 3 > SBR 2	<0.0001	NPI 3 = NPI 2	>0.10
EIF3C				
EIF3D	SBR 2 < SBR 1	<0.0001	NPI 3 < NPI 1	<0.01
	SBR 3 < SBR 1	<0.0001	NPI 2 < NPI 1	<0.10
	SBR 3 = SBR 2	>0.10	NPI 3 = NPI 2	>0.10
EIF3E	SBR 3 > SBR 1	<0.0001	NPI 2 > NPI 1	<0.01
	SBR 3 > SBR 2	<0.0001	NPI 3 = NPI 1	>0.10
	SBR 2 = SBR 1	>0.10	NPI 3 = NPI 2	>0.10
EIF3F	SBR 2 < SBR 1	<0.0001	NPI 2 < NPI 1	<0.0001
	SBR 3 < SBR 1	<0.0001	NPI 3 < NPI 1	<0.0001
	SBR 3 < SBR 2	<0.0001	NPI 3 < NPI 2	<0.01
EIF3G	SBR 3 < SBR 1	<0.0001	NPI 3 < NPI 1	<0.0001
	SBR 3 < SBR 2	<0.0001	NPI 3 < NPI 2	<0.001
	SBR 2 = SBR 1	>0.10	NPI 2 = NPI 1	>0.10
EIF3H	SBR 3 > SBR 1	<0.0001		
	SBR 2 > SBR 1	<0.001		
	SBR 3 = SBR 2	>0.10		
EIF3I	SBR 3 < SBR 1	<0.05	NPI 2 < NPI 1	<0.05
	SBR 2 = SBR 1	>0.10	NPI 3 = NPI 1	>0.10
	SBR 3 = SBR 2	>0.10	NPI 3 = NPI 2	>0.10
EIF3J	SBR 3 > SBR 1	<0.01	NPI 3 > NPI 1	<0.0001
	SBR 2 > SBR 1	<0.05	NPI 3 > NPI 2	<0.0001
	SBR 3 = SBR 2	>0.10	NPI 2 = NPI 1	>0.10
EIF3K	SBR 3 > SBR 1	<0.0001	NPI 3 > NPI 1	<0.0001
	SBR 3 > SBR 2	<0.001	NPI 2 > NPI 1	<0.001
	SBR 2 > SBR 1	<0.01	NPI 3 > NPI 2	<0.10
EIF3L	SBR 2 < SBR 1	<0.0001	NPI 2 < NPI 1	<0.0001
	SBR 3 < SBR 1	<0.0001	NPI 3 < NPI 1	<0.0001
	SBR 3 < SBR 2	<0.0001	NPI 3 < NPI 2	<0.001
EIF3M	SBR 3 > SBR 2	<0.01		
	SBR 3 > SBR 1	<0.05		
	SBR 2 = SBR 1	>0.10		
