# Supplementary material for: Eukaryotic translation initiation factor 3 subunit B could serve as a potential prognostic predictor for breast cancer
Source: Bioengineered. 2022 Jan 18;13(2):2762–76. doi: 10.1080/21655979.2021.2017567 (PMC8974155; doi:10.1080/21655979.2021.2017567)
Supplement: Supplemental Material [file KBIE_A_2017567_SM8035.zip › supplementary/ST2.pdf]

Supplementary Table 2. Survival analyses of the EIF3 complex in all breast cancer.

Gene	Affymetrix ID	Survival outcome	HR	95%CI	P-value
EIF3A	200595_s_at	RFS	1.21	1.09-1.35	**0.00055**
		OS	1.07	0.86-1.32	0.54
		DMFS	0.88	0.73-1.07	0.21
		PPS	1.29	1.01-1.65	**0.04**
	200596_s_at	RFS	1.28	1.15-1.43	**6.90E-06**
		OS	0.87	0.7-1.07	0.18
		DMFS	0.97	0.8-1.18	0.79
		PPS	0.98	0.77-1.24	0.85
	200597_s_at	RFS	0.8	0.72-0.89	**7.00E-05**
		OS	0.62	0.5-0.77	**1.70E-05**
		DMFS	0.74	0.61-0.9	**0.0024**
		PPS	0.91	0.72-1.17	0.47
	210213_s_at	RFS	1.44	1.29-1.61	**5.90E-11**
		OS	1.41	1.14-1.75	**0.0017**
		DMFS	1.16	0.95-1.41	0.13
		PPS	1.08	0.85-1.38	0.54
EIF3B	203462_x_at	RFS	1.29	1.16-1.44	**3.90E-06**
		OS	1.52	1.22-1.88	**0.00014**
		DMFS	1.49	1.22-1.81	**6.10E-05**
		PPS	1.35	1.06-1.73	**0.015**
	208688_x_at	RFS	1.29	1.16-1.44	**3.50E-06**
		OS	1.62	1.3-2.01	**1.20E-05**
		DMFS	1.55	1.28-1.89	**8.90E-06**
		PPS	1.27	1-1.62	0.053
	211501_s_at	RFS	1.46	1.31-1.63	**8.50E-12**
		OS	1.55	1.25-1.93	**6.80E-05**
		DMFS	1.31	1.08-1.59	**0.0068**
		PPS	1.4	1.1-1.79	0.069
EIF3C	200647_x_at	RFS	1.13	1.01-1.25	**0.034**
		OS	0.82	0.66-1.02	0.075
		DMFS	0.94	0.78-1.14	0.54
		PPS	0.76	0.6-0.97	**0.03**
	210949_s_at	RFS	1.12	1.01-1.25	**0.039**
		OS	0.82	0.66-1.01	0.065
		DMFS	0.92	0.76-1.12	0.41
		PPS	0.81	0.63-1.03	0.086
	215230_x_at	RFS	1.29	1.15-1.43	**6.90E-06**
		OS	0.96	0.77-1.19	0.68
		DMFS	1.04	0.85-1.26	0.72
		PPS	0.75	0.59-0.96	**0.02**
EIF3D	200005_at	RFS	1.06	0.95-1.18	0.31
		OS	0.9	0.73-1.12	0.35

		DMFS	0.81	0.67-0.99	**0.036**
		PPS	1.1	0.86-1.4	0.44
EIF3E	208697_s_at	RFS	1.32	1.19-1.48	**4.30E-07**
		OS	1.15	0.93-1.42	0.2
		DMFS	1.06	0.88-1.29	0.54
		PPS	1.13	0.88-1.43	0.34
EIF3F	200023_s_at	RFS	1.13	1.01-1.26	**0.031**
		OS	0.87	0.69-1.11	0.27
		DMFS	0.83	0.69-1.01	0.067
		PPS	0.9	0.73-1.11	0.33
	200865_at	RFS	0.85	0.76-0.94	**0.0026**
		OS	0.91	0.74-1.13	0.4
		DMFS	1.03	0.85-1.25	0.75
		PPS	0.93	0.73-1.19	0.58
EIF3G	208887_at	RFS	0.99	0.89-1.1	0.86
		OS	0.91	0.74-1.13	0.4
		DMFS	0.74	0.61-0.9	**0.027**
		PPS	0.86	0.67-1.1	0.22
EIF3H	201592_at	RFS	1.18	1.05-1.31	**0.0036**
		OS	0.88	0.71-1.09	0.25
		DMFS	0.97	0.8-1.18	0.8
		PPS	0.72	0.56-0.92	**0.0083**
EIF3I	208756_at	RFS	0.87	0.78-0.97	**0.015**
		OS	0.84	0.68-1.04	0.12
		DMFS	0.81	0.67-0.98	**0.03**
		PPS	0.84	0.65-1.07	0.15
EIF3J	208264_s_at	RFS	1.18	1.06-1.32	**0.0022**
		OS	1.21	0.98-1.5	0.076
		DMFS	0.98	0.81-1.19	0.85
		PPS	1.14	0.9-1.46	0.28
	208985_s_at	RFS	1.49	1.33-1.66	**9.40E-13**
		OS	1.45	1.17-1.8	**0.00068**
		DMFS	1.14	0.94-1.39	0.17
		PPS	1.12	0.88-1.43	0.36
EIF3K	210501_x_at	RFS	1.11	0.99-1.23	0.071
		OS	1.06	0.86-1.32	0.57
		DMFS	0.93	0.77-1.13	0.48
		PPS	0.85	0.66-1.08	0.18
	212716_s_at	RFS	1.27	1.14-1.42	**1.40E-05**
		OS	1.18	0.95-1.46	0.14
		DMFS	0.94	0.78-1.14	0.54
		PPS	1.12	0.88-1.43	0.36
	221494_x_at	RFS	1.18	1.06-1.32	**0.0028**
		OS	1.08	0.87-1.35	0.46

		DMFS	0.96	0.79-1.17	0.71
		PPS	0.84	0.65-1.07	0.15
EIF3L	217719_at	RFS	1.08	0.97-1.2	0.16
		OS	0.94	0.76-1.17	0.59
		DMFS	0.78	0.65-0.95	**0.014**
		PPS	0.96	0.75-1.22	0.74
EIF3M	202231_at	RFS	1.5	1.34-1.67	**3.80E-13**
		OS	0.94	0.76-1.17	0.59
		DMFS	1.15	0.95-1.39	0.16
		PPS	1.48	1.16-1.89	**0.0017**
	202232_s_at	RFS	1.42	1.27-1.59	**2.30E-10**
		OS	1.12	0.91-1.39	0.29
		DMFS	1.18	0.97-1.43	0.095
		PPS	1.51	1.18-1.93	**0.00091**
	215190_at	RFS	0.76	0.68-0.85	**1.00E-06**
		OS	1.09	0.88-1.34	0.45
		DMFS	0.95	0.78-1.15	0.6
		PPS	0.92	0,72-1.17	0.49
