# Supplementary material for: Eukaryotic translation initiation factor 3 subunit B could serve as a potential prognostic predictor for breast cancer
Source: Bioengineered. 2022 Jan 18;13(2):2762–76. doi: 10.1080/21655979.2021.2017567 (PMC8974155; doi:10.1080/21655979.2021.2017567)
Supplement: Supplemental Material [file KBIE_A_2017567_SM8035.zip › supplementary/ST3.pdf]

**Supplementary Table 3. Survival analyses of the EIF3 complex with different lymph node status in all breast cancer.**

| Gene | Affymetrix ID | Survival outcome | Lymph node positive | | | Lymph node negative | | |
|---|---|---|---|---|---|---|---|---|
| | | | HR | 95%CI | P-value | HR | 95%CI | P-value |
| ***EIF3A*** | 200595_s_at | RFS | 1 | 0.82-1.21 | 0.97 | 0.95 | 0.8-1.12 | 0.53 |
| | | OS | 0.85 | 0.58-1.26 | 0.42 | 0.96 | 0.66-1.39 | 0.84 |
| | | DMFS | 0.79 | 0.53-1.16 | 0.22 | 0.91 | 0.69-1.19 | 0.49 |
| | | PPS | 0.49 | 0.31-0.77 | **0.0018** | 1.29 | 1.01-1.65 | **0.04** |
| | 200596_s_at | RFS | 0.96 | 0.79-1.17 | 0.71 | 1 | 0.85-1.19 | 0.97 |
| | | OS | 0.91 | 0.62-1.34 | 0.62 | 0.71 | 0.49-1.03 | 0.073 |
| | | DMFS | 1.34 | 0.91-1.98 | 0.13 | 0.88 | 0.67-1.15 | 0.35 |
| | | PPS | 1.01 | 0.64-1.58 | 0.98 | 0.98 | 0.64-1.5 | 0.93 |
| | 200597_s_at | RFS | 0.81 | 0.66 | **0.033** | 0.93 | 0.78-1.1 | 0.38 |
| | | OS | 0.72 | 0.49-1.07 | 0.11 | 0.79 | 0.55-1.15 | 0.22 |
| | | DMFS | 0.77 | 0.53-1.14 | 0.19 | 0.93 | 0.71-1.22 | 0.62 |
| | | PPS | 0.9 | 0.57-1.4 | 0.63 | 0.88 | 0.58-1.35 | 0.56 |
| | 210213_s_at | RFS | 1.18 | 0.97-1.43 | 0.1 | 1.37 | 1.15-1.62 | **0.00029** |
| | | OS | 1.37 | 0.93-2.02 | 0.11 | 1.99 | 1.34-2.94 | **0.00045** |
| | | DMFS | 1.15 | 0.78-1.69 | 0.48 | 1.25 | 0.95-1.64 | 0.11 |
| | | PPS | 1.49 | 0.95-2.34 | 0.079 | 1.13 | 0.73-1.73 | 0.59 |
| ***EIF3B*** | 203462_x_at | RFS | 1.61 | 1.32-1.97 | **2.10E-06** | 1.24 | 1.04-1.46 | **0.013** |
| | | OS | 1.48 | 1-2.19 | **0.047** | 1.14 | 0.79-1.65 | 0.49 |
| | | DMFS | 1.54 | 1.04-2.27 | **0.03** | 1.2 | 0.92-1.58 | 0.18 |
| | | PPS | 1.83 | 1.17-2.89 | **0.0078** | 1.08 | 0.71-1.66 | 0.71 |
| | 208688_x_at | RFS | 1.72 | 1.41-2.1 | **7.10E-08** | 1.26 | 1.06-1.49 | **0.008** |
| | | OS | 1.43 | 0.97-2.11 | 0.072 | 1.16 | 0.8-1.69 | 0.43 |
| | | DMFS | 1.54 | 1.04-2.27 | **0.03** | 1.16 | 0.89-1.52 | 0.27 |
| | | PPS | 1.59 | 1.01-2.49 | **0.043** | 0.99 | 0.64-1.52 | 0.95 |
| | 211501_s_at | RFS | 1.22 | 1.01-1.49 | **0.043** | 1.19 | 1-1.41 | **0.045** |
| | | OS | 1.7 | 1.14-2.52 | **0.0077** | 1.59 | 1.08-2.35 | **0.017** |
| | | DMFS | 1.67 | 1.13-2.47 | **0.0099** | 1.25 | 0.95-1.64 | 0.11 |
| | | PPS | 1.35 | 0.86-2.12 | 0.19 | 1.36 | 0.88-2.08 | 0.16 |
| ***EIF3C*** | 200647_x_at | RFS | 0.92 | 0.76-1.12 | 0.42 | 1.1 | 0.93-1.31 | 0.26 |
| | | OS | 0.96 | 0.66-1.42 | 0.86 | 0.99 | 0.68-1.45 | 0.96 |
| | | DMFS | 0.89 | 0.6-1.31 | 0.56 | 0.96 | 0.73-1.26 | 0.77 |
| | | PPS | 1.01 | 0.64-1.57 | 0.98 | 0.83 | 0.54-1.27 | 0.39 |
| | 210949_s_at | RFS | 0.87 | 0.72-1.06 | 0.17 | 1.05 | 0.89-1.24 | 0.58 |
| | | OS | 1.04 | 0.7-1.52 | 0.86 | 0.77 | 0.53-1.11 | 0.16 |
| | | DMFS | 0.94 | 0.64-1.38 | 0.75 | 0.94 | 0.72-1.24 | 0.67 |
| | | PPS | 1.21 | 0.77-1.9 | 0.4 | 0.71 | 0.46-1.08 | 0.11 |
| | 215230_x_at | RFS | 1.02 | 0.84-1.24 | 0.84 | 1.21 | 1.02-1.44 | **0.025** |
| | | OS | 1.17 | 0.79-1.72 | 0.44 | 1.09 | 0.74-1.59 | 0.66 |
| | | DMFS | 0.94 | 0.64-1.38 | 0.76 | 1.02 | 0.78-1.34 | 0.9 |
| | | PPS | 0.78 | 0.5-1.22 | 0.28 | 0.92 | 0.6-1.41 | 0.71 |

| | | | | | | | | |
|---|---|---|---|---|---|---|---|---|
| ***EIF3D*** | 200005_at | RFS | 0.95 | 0.78-1.15 | 0.58 | 0.85 | 0.72-1.01 | 0.061 |
| | | OS | 1.18 | 0.8-1.74 | 0.39 | 0.78 | 0.54-1.13 | 0.19 |
| | | DMFS | 1.48 | 1-2.19 | **0.047** | 0.68 | 0.52-0.89 | **0.0054** |
| | | PPS | 1.12 | 0.72-1.76 | 0.61 | 0.92 | 0.6-1.41 | 0.7 |
| ***EIF3E*** | 208697_s_at | RFS | 1.11 | 0.91-1.35 | 0.29 | 1.2 | 1.01-1.42 | **0.035** |
| | | OS | 1.06 | 0.72-1.57 | 0.76 | 0.98 | 0.68-1.42 | 0.92 |
| | | DMFS | 1.33 | 0.9-1.97 | 0.14 | 0.93 | 0.71-1.22 | 0.6 |
| | | PPS | 1.06 | 0.68-1.66 | 0.79 | 1.16 | 0.76-1.78 | 0.49 |
| ***EIF3F*** | 200023_s_at | RFS | 0.91 | 0.75-1.11 | 0.34 | 1.02 | 0.86-1.21 | 0.81 |
| | | OS | 0.83 | 0.57-1.23 | 0.36 | 1.01 | 0.7-1.46 | 0.96 |
| | | DMFS | 0.67 | 0.45-0.99 | **0.041** | 0.84 | 0.64-1.11 | 0.22 |
| | | PPS | 0.62 | 0.39-0.97 | **0.035** | 0.96 | 0.63-1.48 | 0.87 |
| | 200865_at | RFS | 1 | 0.82-1.22 | 0.99 | 0.85 | 0.72-1 | 0.054 |
| | | OS | 1.01 | 0.69-1.49 | 0.95 | 0.76 | 0.53-1.11 | 0.16 |
| | | DMFS | 1.35 | 0.91-1.98 | 0.13 | 0.9 | 0.68-1.18 | 0.44 |
| | | PPS | 1.4 | 0.9-2.2 | 0.14 | 0.7 | 0.45-1.08 | 0.11 |
| ***EIF3G*** | 208887_at | RFS | 0.84 | 0.69-1.03 | 0.087 | 0.88 | 0.75-1.05 | 0.15 |
| | | OS | 1.16 | 0.78-1.7 | 0.46 | 1.09 | 0.75-1.59 | 0.66 |
| | | DMFS | 0.57 | 0.38-0.84 | **0.0042** | 0.73 | 0.56-1.69 | **0.025** |
| | | PPS | 0.96 | 0.62-1.51 | 0.87 | 1.09 | 0.71-1.67 | 0.69 |
| ***EIF3H*** | 201592_at | RFS | 0.94 | 0.77-1.14 | 0.53 | 1.12 | 0.95-1.33 | 0.18 |
| | | OS | 0.83 | 0.57-1.23 | 0.36 | 1.15 | 0.79-1.67 | 0.47 |
| | | DMFS | 0.88 | 0.6-1.3 | 0.52 | 1.14 | 0.87-1.49 | 0.35 |
| | | PPS | 0.67 | 0.43-1.05 | 0.076 | 0.86 | 0.56-1.32 | 0.49 |
| ***EIF3I*** | 208756_at | RFS | 1.01 | 0.83-1.23 | 0.92 | 0.83 | 0.7-0.99 | **0.033** |
| | | OS | 1 | 0.68-1.48 | 0.99 | 0.86 | 0.59-1.24 | 0.41 |
| | | DMFS | 1.04 | 0.71-1.53 | 0.83 | 0.8 | 0.61-1.04 | 0.098 |
| | | PPS | 0.97 | 0.62-1.52 | 0.89 | 0.9 | 0.59-1.39 | 0.64 |
| ***EIF3J*** | 208264_s_at | RFS | 0.93 | 0.76-1.13 | 0.44 | 1.06 | 0.89-1.25 | 0.5 |
| | | OS | 0.92 | 0.62-1.36 | 0.68 | 1.33 | 0.91-1.92 | 0.14 |
| | | DMFS | 0.88 | 0.6-1.3 | 0.52 | 1.01 | 0.77-1.33 | 0.93 |
| | | PPS | 0.64 | 0.41-1.01 | 0.051 | 1.06 | 0.69-1.62 | 0.79 |
| | 208985_s_at | RFS | 1.01 | 0.83-1.23 | 0.93 | 1.31 | 1.11-1.55 | **0.0016** |
| | | OS | 0.99 | 0.67-1.46 | 0.96 | 0.98 | 1.35-2.9 | **0.00038** |
| | | DMFS | 1.11 | 0.75-1.63 | 0.61 | 1.15 | 0.87-1.5 | 0.33 |
| | | PPS | 0.79 | 0.5-1.23 | 0.29 | 1.38 | 0.9-2.12 | 0.14 |
| ***EIF3K*** | 210501_x_at | RFS | 1.09 | 0.9 | 1.33 | 0.97 | 0.82-1.15 | 0.71 |
| | | OS | 1.27 | 0.86-1.87 | 0.22 | 1.03 | 0.71-1.49 | 0.9 |
| | | DMFS | 1.16 | 0.79-1.71 | 0.44 | 0.89 | 0.68-1.17 | 0.42 |
| | | PPS | 1.42 | 0.9-2.22 | 0.13 | 1.07 | 0.7-1.64 | 0.75 |
| | 212716_s_at | RFS | 1.11 | 0.91-1.35 | 0.31 | 0.97 | 0.82-1.15 | 0.71 |
| | | OS | 1.46 | 0.99-2.16 | 0.057 | 1.07 | 0.74-1.55 | 0.73 |
| | | DMFS | 0.96 | 0.66-1.42 | 0.85 | 0.92 | 0.7-1.2 | 0.54 |
| | | PPS | 0.82 | 0.53-1.29 | 0.39 | 1.1 | 0.71-1.68 | 0.67 |

| | | | | | | | | |
|---|---|---|---|---|---|---|---|---|
| | 221494_x_at | RFS | 1.09 | 0.89-1.32 | 0.4 | 1.04 | 0.88-1.23 | 0.62 |
| | | OS | 1.54 | 1.04-2.27 | **0.03** | 1.15 | 0.79-1.68 | 0.47 |
| | | DMFS | 1.15 | 0.78-1.69 | 0.48 | 0.92 | 0.7-1.2 | 0.54 |
| | | PPS | 1.4 | 0.9-2.19 | 0.14 | 1.17 | 0.76-1.79 | 0.48 |
| ***EIF3L*** | 217719_at | RFS | 0.88 | 0.73-1.07 | 0.21 | 0.89 | 0.75-1.05 | 0.17 |
| | | OS | 1.17 | 0.8-1.73 | 0.42 | 1.07 | 0.75-1.55 | 0.72 |
| | | DMFS | 0.84 | 0.57-1.24 | 0.37 | 0.77 | 0.59-1.01 | 0.063 |
| | | PPS | 0.69 | 0.44-1.08 | 0.11 | 1.27 | 0.83-1.94 | 0.27 |
| ***EIF3M*** | 202231_at | RFS | 1.07 | 0.88-1.31 | 0.48 | 1.2 | 1.01-1.42 | **0.033** |
| | | OS | 1.04 | 0.71-1.53 | 0.84 | 2.08 | 1.42-3.04 | **0.00012** |
| | | DMFS | 1.27 | 0.86-1.88 | 0.22 | 1.24 | 0.95-1.23 | 0.122 |
| | | PPS | 0.53 | 0.34-0.83 | **0.0053** | 2.26 | 1.46-3.5 | **0.00018** |
| | 202232_s_at | RFS | 0.91 | 0.75-1.11 | 0.37 | 1.2 | 1.01-1.42 | **0.037** |
| | | OS | 0.81 | 0.55-1.19 | 0.28 | 1.73 | 1.18-2.52 | **0.0042** |
| | | DMFS | 0.93 | 0.63-1.38 | 0.73 | 1.28 | 0.98-1.68 | 0.074 |
| | | PPS | 0.61 | 0.39-0.96 | **0.033** | 1.39 | 0.9-2.13 | 0.13 |
| | 215190_at | RFS | 0.97 | 0.8-1.18 | 0.78 | 0.97 | 0.82-1.15 | 0.72 |
| | | OS | 0.81 | 0.55-1.19 | 0.28 | 0.68 | 0.46-1 | **0.049** |
| | | DMFS | 1.03 | 0.7-1.51 | 0.88 | 0.82 | 0.62-1.07 | 0.14 |
| | | PPS | 1.43 | 0.91-2.24 | 0.11 | 0.86 | 0.56-1.32 | 0.48 |
