# Supplementary material for: Eukaryotic translation initiation factor 3 subunit B could serve as a potential prognostic predictor for breast cancer
Source: Bioengineered. 2022 Jan 18;13(2):2762–76. doi: 10.1080/21655979.2021.2017567 (PMC8974155; doi:10.1080/21655979.2021.2017567)
Supplement: Supplemental Material [file KBIE_A_2017567_SM8035.zip › supplementary/ST4.pdf]

Supplementary Table 4. Survival analyses of the EIF3 complex with different molecular subtypes based on the 2013 St Gallen criteria in all breast cancer.

			Grade I		Grade II		Grade III	
**Gene**	**Affymetrix ID**	**Survival outcome**	**HR（95%CI）**	**P-value**	**HR（95%CI）**	**P-value**	**HR（95%CI）**	**P-value**
EIF3A	200595_s_at	RFS	1（0.82-1.21）	0.66	0.76（0.6-0.97）	**0.026**	0.95（0.67-1.34）	0.76
		OS	1.13（0.46-2.78）	0.78	0.86（0.56-1.33）	0.5	1.05（0.76-1.46）	0.77
		DMFS	0.83（0.37-0.89）	0.66	0.89（0.63-1.25）	0.49	0.95（0.67-1.34）	0.76
		PPS	1.24（0.46-3.31）	0.67	0.91（0.56-1.47）	0.69	0.92（0.63-1.34）	0.66
	200596_s_at	RFS	1.46（0.86-2.47）	0.16	1.19（0.93-1.51）	0.17	0.98（0.79-1.22）	0.88
		OS	0.98（0.64-1.5）	0.92	0.98（0.64-1.5）	0.92	0.77（0.55-1.07）	0.12
		DMFS	1（0.71-1.4）	0.98	1（0.71-1.4）	0.98	0.98（0.7-1.39）	0.92
		PPS	1.06（0.66-1.72）	0.81	1.06（0.66-1.72）	0.81	1.01（0.69-1.47）	0.97
	200597_s_at	RFS	1.24（0.74-2.09）	0.41	0.75（0.59-0.95）	**0.017**	1.07（0.86-1.33）	0.53
		OS	0.76（0.3-1.92）	0.56	0.74（0.48-1.14）	0.17	0.79（0.57-1.1）	0.16
		DMFS	0.81（0.35-1.88）	0.62	0.77（0.55-1.1）	0.15	1.28（0.91-1.82）	0.16
		PPS	0.91（0.33-2.48）	0.85	1.26（0.78-2.05）	0.35	0.79（0.54-1.16）	0.24
	210213_s_at	RFS	1.2（0.71-2.03）	0.49	1.22（0.96-1.56）	0.1	0.95（0.76-1.18）	0.64
		OS	0.82（0.67-4.94）	0.23	1.51（0.98-2.34）	0.062	1.1（0.79-1.53）	57
		DMFS	1.95（0.8-4.79）	0.14	1.19（0.84-1.67）	0.33	1.14（0.81-1.62）	0.45
		PPS	2.68（0.91-7.9）	0.063	0.96（0.65-1.4）	0.82	0.96（0.65-1.4）	0.82
EIF3B	203462_x_at	RFS	1.79（1.04-3.1）	**0.033**	1.21（0.95-1.55）	0.11	1.28（1.03-1.59）	**0.025**
		OS	1.1（0.41-2.91）	0.85	1.85（1.19-2.88）	**0.0058**	1.15（0.83-1.59）	0.41
		DMFS	2.68（1.05-6.85）	**0.033**	1.45（1.03-2.06）	**0.034**	1.13（0.8-1.59）	0.49
		PPS	0.82（0.3-2.2）	0.69	1.72（1.05-2.88）	**0.028**	1.03（0.7-1.51）	0.88
	208688_x_at	RFS	1.59（0.93-2.71）	0.087	1.28（1.01-1.63）	**0.042**	1.48（1.19-1.84）	**0.00041**
		OS	0.72（0.28-1.85）	0.49	1.91（1.22-2.98）	**0.0038**	1.24（0.9-1.73）	0.19
		DMFS	1.51（0.64-3.53）	0.34	1.48（1.05-2.1）	**0.025**	1.26（0.89-1.78）	0.19
		PPS	0.4（0.14-1.15）	0.077	1.6（0.98-2.6）	0.058	1.19（0.81-1.74）	0.38
	211501_s_at	RFS	1.03（0.61-1.73）	0.91	1.13（0.89-1.44）	0.31	1.12（0.9-1.39）	0.31
		OS	0.93（0.37-2.33）	0.88	1.36（0.88-2.11）	0.16	1.3（0.93-1.8）	0.12
		DMFS	0.81（0.35-1.88）	0.62	1.05（0.74-1.48）	0.78	1.15（0.81-1.62）	0.44
		PPS	0.81（0.3-2.21）	0.68	0.81（0.3-2.21）	0.68	1.27（0.86-1.85）	0.22
EIF3C	200647_x_at	RFS	0.83（0.5-1.4）	0.49	1.05（0.83-0.34）	0.67	1.32（1.06-1.64）	**0.013**
		OS	0.94（0.39-2.28）	0.89	0.8（0.52-1.24）	0.32	1.03（0.74-1.44）	0.85
		DMFS	0.69（0.3-1.59）	0.38	1（0.71-1.41）	1	1.27（0.89-1.8）	0.18
		PPS	1.38（0.48-3.99）	0.55	0.79（0.49-1.28）	0.34	0.91（0.61-1.33）	0.61
	210949_s_at	RFS	1.15（0.68-1.94）	0.61	0.99（0.78-1.26）	0.93	1.1（0.89-1.37）	0.38
		OS	0.88（0.36-2.13）	0.77	0.88（0.58-1.36）	0.57	0.96（0.69-1.33）	0.79
		DMFS	0.86（0.37-2）	0.73	0.96（0.68-1.35）	0.81	0.85（0.6-1.2）	0.34
		PPS	0.86（0.3-2.47）	0.78	1.06（0.65-1.72）	0.82	0.88（0.6-1.29）	0.51
	215230_x_at	RFS	0.79（0.47-1.34）	0.39	1.21（0.95-1.54）	0.13	1.21（0.97-1.51）	0.086
		OS	0.38（0.14-0.98）	**0.037**	1.02（0.66-1.58）	0.92	1.15（0.83-1.6）	0.39
		DMFS	0.53（0.23-1.22）	0.13	0.99（0.7-1.4）	0.95	1.42（1-2.02）	**0.049**

		PPS	0.63（0.22-1.82）	0.39	0.58（0.36-0.95）	**0.028**	0.87（0.6-1.28）	0.49
EIF3D	200005_at	RFS	0.57（0.33-0.98）	**0.039**	0.92（0.72-1.16）	0.47	0.91（0.73-1.13）	0.38
		OS	0.87（0.35-2.15）	0.77	0.79（0.51-1.21）	0.28	1.11（0.8-1.55）	0.52
		DMFS	0.4（0.16-1.02）	**0.048**	0.97（0.69-1.36）	0.85	0.77（0.54-1.09）	0.14
		PPS	0.91（0.34-2.15）	0.86	0.8（0.49-1.3）	0.36	1.52（1.03-2.23）	**0.032**
EIF3E	208697_s_at	RFS	0.73（0.43-1.23）	0.24	0.98（0.77-1.250	0.87	1（0.8-1.24）	0.99
		OS	0.6（0.24-1.47）	0.26	0.72（0.47-1.1）	0.13	0.92（0.66-1.28）	0.62
		DMFS	0.58（0.25-1.34）	0.19	0.93（0.66-1.31）	0.68	0.94（0.67-1.33）	0.73
		PPS	0.92（0.34-2.48）	0.87	0.75（0.46-1.23）	0.25	1.3（0.89-1.89）	0.18
EIF3F	200023_s_at	RFS	0.86（0.51-1.44）	0.56	0.95（0.75-1.21）	0.68	1.12（0.9-1.39）	0.31
		OS	0.8（0.32-2.01）	0.64	0.75（0.49-1.15）	0.19	1（0.72-1.39）	1
		DMFS	0.97（0.43-2.21）	0.94	0.95（0.68-1.34）	0.79	0.96（0.68-1.36）	0.82
		PPS	1.42（0.53-3.83）	0.48	0.48（0.29-0.79）	**0.003**	1.03（0.7-1.5）	0.89
	200865_at	RFS	0.91（0.54-1.53）	0.72	0.97（0.76-1.23）	0.81	0.95（0.76-1.18）	0.65
		OS	0.52（0.19-1.4）	0.19	1.13（0.73-1.74）	0.58	1.14（0.82-1.59）	0.42
		DMFS	0.4（0.17-0.96）	**0.034**	0.72（0.51-1.02）	0.064	0.75（0.53-1.06）	0.098
		PPS	0.37（0.13-1.04）	0.052	0.73（0.45-1.18）	0.2	1.24（0.85-1.82）	0.26
EIF3G	208887_at	RFS	0.91（0.54-1.53）	0.72	0.97（0.76-1.23）	0.81	0.95（0.76-1.18）	0.65
		OS	0.52（0.19-1.4）	0.19	1.13（0.73-1.74）	0.58	1.14（0.82-1.59）	0.42
		DMFS	0.4（0.17-0.96）	**0.034**	0.72（0.51-1.02）	0.064	0.75（0.53-1.06）	0.098
		PPS	0.37（0.13-1.04）	0.052	0.73（0.45-1.18）	0.2	1.24（0.85-1.82）	0.26
EIF3H	201592_at	RFS	1.14（0.68-1.92）	0.61	0.89（0.7-1.13）	0.34	1.03（0.82-1.27）	0.82
		OS	1.43（0.59-3.51）	0.44	0.73（0.48-1.13）	0.16	0.97（0.7-1.35）	0.88
		DMFS	0.92（0.41-2.1）	0.85	0.86（0.61-1.22）	0.4	1.07（0.75-1.51）	0.72
		PPS	1.56（0.57-4.25）	0.38	0.56（0.34-0.92）	**0.021**	0.82（0.56-1.2）	0.31
EIF3I	208756_at	RFS	0.67（0.25-1.85）	0.44	0.81（0.64-1.03）	0.082	0.87（0.7-1.09）	0.22
		OS	0.72（0.43-1.22）	0.23	0.74（0.48-1.14）	0.18	1.04（0.75-1.44）	0.83
		DMFS	0.71（0.31-1.64）	0.42	0.75（0.53-1.07）	0.11	0.84（0.59-1.19）	0.32
		PPS	0.78（0.32-1.89）	0.58	0.75（0.46-1.23）	0.25	1.01（0.68-1.48）	0.97
EIF3J	208264_s_at	RFS	0.86（0.51-1.45）	0.57	0.92（0.72-1.17）	0.51	1.02（0.82-1.27）	0.87
		OS	0.74（0.29-1.87）	0.52	1.39（0.9-2.13）	0.13	1.05（0.76-1.46）	0.77
		DMFS	0.99（0.42-2.33）	0.99	0.89（0.63-1.26）	0.51	1.15（0.81-1.63)	0.43
		PPS	0.51（0.15-1.5）	0.22	1.14（0.7-1.85）	0.59	0.79（0.54-1.16）	0.22
	208985_s_at	RFS	1.03（0.62-1.73）	0.9	1.17（0.92-1.49）	0.2	1.14（0.92-1.42）	0.22
		OS	2.15（0.82-5.63）	0.11	1.48（0.96-2.27）	0.076	1.56（1.12-2.16）	**0.0082**
		DMFS	1.29（0.56-2.93）	0.55	0.98（0.7-1.38）	0.91	1.09（0.77-1.54）	0.64
		PPS	1.36（0.51-3.63）	0.54	1.12（0.69-1.82）	0.64	1.22（0.83-1.79）	0.3
EIF3K	210501_x_at	RFS	0.94（0.56-1.58）	0.81	1.02（0.8-1.3）	0.86	1.04（0.84-1.29）	0.72
		OS	1.74（0.64-4.71）	0.27	1.11（0.72-1.7）	0.64	0.93（0.67-1.3）	0.68
		DMFS	0.86（0.37-1.96）	0.72	0.86（0.61-1.21）	0.38	0.88（0.63-1.25）	0.49
		PPS	1.49（0.55-4.08）	0.43	0.77（0.47-1.26）	0.3	0.87（0.6-1.28）	0.49
	212716_s_at	RFS	0.82（0.49-1.39）	0.46	1.06（0.84-1.35）	0.61	1.25（1-1.55）	**0.047**
		OS	1.94（0.77-4.9)	0.15	0.91（0.59-1.39）	0.66	1.37（0.99-1.9）	0.059
		DMFS	0.73（0.32-1.66）	0.45	0.82（0.58-1.16）	0.26	0.92（0.65-1.29）	0.62

		PPS	2.57（0.87-7.54）	0.076	0.71（0.44-1.16）	0.17	1.03（0.71-1.51）	0.87
	221494_x_at	RFS	0.95（0.56-1.6）	0.85	1（0.78-1.27）	0.98	1（0.81-1.25）	0.97
		OS	0.91（0.35-2.36）	0.85	0.87（0.56-1.33）	0.51	1.04（0.75-1.45）	0.8
		DMFS	1.02（0.43-2.44）	0.96	0.8（0.56-1.12）	0.19	0.91（0.64-1.29）	0.59
		PPS	1.15（0.43-3.12）	0.78	0.68（0.42-1.11）	0.12	0.95（0.65-1.4）	0.8
EIF3L	217719_at	RFS	0.73（0.43-1.23）	0.23	0.84（0.66-1.07）	0.15	1.06（0.85-1.31）	0.62
		OS	0.81（0.33-1.99）	0.65	0.63（0.41-0.98）	**0.038**	1.05（0.76-1.46）	0.76
		DMFS	0.37（0.15-0.89）	**0.022**	0.73（0.52-1.03）	0.076	0.95（0.67-1.34）	0.77
		PPS	0.72（0.27-1.94）	0.51	0.55（0.34-0.91）	**0.017**	1.01（0.69-1.48）	0.97
EIF3M	202231_at	RFS	0.97（0.58-1.63）	0.91	1.07（0.85-1.37）	0.55	1.06（0.72-1.55）	0.58
		OS	1.04（0.42-2.6）	0.93	1.37（0.89-2.1）	0.15	1.49（1.07-2.07）	**0.017**
		DMFS	0.75（0.33-1.72）	0.5	1.27（0.9-1.79）	0.18	0.94（0.66-1.33）	0.73
		PPS	0.83（0.31-2.23）	0.71	1.44（0.88-2.34）	0.14	1.06（0.85-1.32）	0.78
	202232_s_at	RFS	1.19（0.7-2.01）	0.52	0.99（0.78-1.26）	0.93	0.97（0.78-1.2）	0.77
		OS	1.13（0.44-2.89）	0.79	0.99（0.65-1.51）	0.96	1.05（0.75-1.46）	0.78
		DMFS	0.68（0.29-1.61）	0.38	0.94（0.66-1.32）	0.7	0.87（0.62-1.24）	0.45
		PPS	0.94（0.35-2.56）	0.91	0.72（0.44-1.17）	0.19	0.97（0.66-1.42）	0.87
	215190_at	RFS	1（0.6-1.69）	0.99	1.12（0.88-1.42）	0.36	1.09（0.88-1.35）	0.44
		OS	0.69（0.26-1.82）	0.46	1（0.65-1.54）	0.99	1.13（0.82-1.57）	0.46
		DMFS	1.15（0.49-2.71）	0.74	1.1（0.78-1.56）	0.57	1.05（0.74-1.48）	0.78
		PPS	0.62（0.22-1.74）	0.36	0.96（0.59-1.55）	0.85	1.03（0.7-1.5）	0.89
